# Long-Term Temperature Stress in the Coral Model Aiptasia Supports the “Anna Karenina Principle” for Bacterial Microbiomes

**DOI:** 10.3389/fmicb.2019.00975

**Published:** 2019-05-08

**Authors:** Hanin Ibrahim Ahmed, Marcela Herrera, Yi Jin Liew, Manuel Aranda

**Affiliations:** Division of Biological and Environmental Science and Engineering, Red Sea Research Center, King Abdullah University of Science and Technology, Thuwal, Saudi Arabia

**Keywords:** β-diversity, dispersion, microbiome, 16S rRNA gene, temperature

## Abstract

The understanding of host-microbial partnerships has become a hot topic during the last decade as it has been shown that associated microbiota play critical roles in the host physiological functions and susceptibility to diseases. Moreover, the microbiome may contribute to host resilience to environmental stressors. The sea anemone Aiptasia is a good laboratory model system to study corals and their microbial symbiosis. In this regard, studying its bacterial microbiota provides a better understanding of cnidarian metaorganisms as a whole. Here, we investigated the bacterial communities of different Aiptasia host-symbiont combinations under long-term heat stress in laboratory conditions. Following a 16S rRNA gene sequencing approach we were able to detect significant differences in the bacterial composition and structure of Aiptasia reared at different temperatures. A higher number of taxa (i.e., species richness), and consequently increased α-diversity and β-dispersion, were observed in the microbiomes of heat-stressed individuals across all host strains and experimental batches. Our findings are in line with the recently proposed Anna Karenina principle (AKP) for animal microbiomes, which states that dysbiotic or stressed organisms have a more variable and unstable microbiome than healthy ones. Microbial interactions affect the fitness and survival of their hosts, thus exploring the AKP effect on animal microbiomes is important to understand host resilience. Our data contributes to the current knowledge of the Aiptasia holobiont and to the growing field of study of host-associated microbiomes.

## Introduction

Corals and their association with different microbes, such as photosynthetic algae from the family Symbiodiniaceae (Lajeunesse et al., 2018) and diverse bacterial communities (Ritchie, 2006; Rosenberg et al., 2007; Ainsworth et al., 2014), is one of the most studied symbioses. The symbiotic microbiota has an important role in the fitness and survival of corals (Bourne et al., 2016), and deviations from a community equilibrium might lead to disease and even death (Bourne et al., 2008; Cárdenas et al., 2012; Closek et al., 2014). Hence, studying the interactions between the host, microbiome and environment is crucial to better comprehend the susceptibility and resilience of coral holobionts to environmental perturbations (Bourne et al., 2008; Kelly et al., 2014; Röthig et al., 2016b; Ziegler et al., 2016; Chen et al., 2017).

Increases in sea surface temperature of just a few degrees can lead to coral bleaching (Hoegh-Guldberg, 1999) and increased susceptibility to disease (Bruno et al., 2007; Harvell et al., 2015; Maynard et al., 2015). Elevated temperatures alter the structure and diversity of the coral-associated microbiota, in many cases resulting in the proliferation of opportunistic bacteria (Ritchie, 2006; Rosenberg et al., 2007; Bourne et al., 2009; Mao-Jones et al., 2010). Even when caused by short-term exposure to high temperatures, thermal stress can cause rapid and long-term shifts in the microbial communities of corals that ultimately favor pathogenic states (Bourne et al., 2009). Similarly, changes in microbiota also occur under acidification, pollution and overfishing stress (Jessen et al., 2013; McDevitt-Irwin et al., 2017). Further, coral responses to heat stress are not ubiquitous; some species can be severely affected (Carpenter et al., 2008; Hoogenboom et al., 2017; Scheufen et al., 2017) while others might be able to persist (Hoogenboom et al., 2017). It is still not fully known what drives resilience but evidence of shifts, or lack thereof, in the microbial composition of heat-stressed corals strongly suggests that microbiota may be a crucial component of coral resilience (Bourne et al., 2008; Lee et al., 2015; Hadaidi et al., 2017).

Corals are, however, notoriously difficult to maintain in aquariums and studying them *in situ* is not always possible, which makes lab-based molecular and some ecological work challenging (Weis et al., 2008; Voolstra, 2013). Instead, the sea anemone Aiptasia (*sensu Exaiptasia pallida*, Grajales and Rodríguez, 2014) has been used to study cnidarian-microbial interactions. Among its advantages are that it grows fast, produces large clonal populations, is able to live in a symbiont-free state and that it can be re-infected with different Symbiodiniaceae types under laboratory conditions (Weis et al., 2008; Voolstra, 2013). Furthermore, two distinct populations have been identified based on their endosymbiotic relationship with different Symbiodiniaceae species (Thornhill et al., 2013); H2, a globally distributed lineage (found in Hawaii, Japan, the Mediterranean, and Australia) and CC7, a local lineage (found exclusively in Florida). Recent studies have also investigated their bacterial associations in wild (Brown et al., 2017) and laboratory settings (Röthig et al., 2016a; Herrera et al., 2017). The latter has allowed to culture pure bacterial isolates (Röthig et al., 2016a), thus providing a promising foundation for future functional studies that intend to identify bacteria that are critical for environmental resilience of the holobiont, such as increased thermotolerance.

Here, we assessed changes in the bacterial communities associated with four different Aiptasia host-symbiont combinations across two temperatures (25°C and 32°C) based on a 16S rRNA gene sequencing approach. Moreover, we studied the effect of long-term heat stress on the bacterial diversity and dispersion while taking into account variation resulting from the biological differences between Aiptasia groups and technical replicates (i.e., batch effects).

## Materials and Methods

### Experimental Set-Up

Symbiotic anemones from three different clonal Aiptasia strains were used in this study; H2 from Hawaii, CC7 from North Carolina and a Red Sea (RS) line collected from the Saudi Arabian coast of the southern RS. These, at the same time, associate with specific Symbiodiniaceae species (Thornhill et al., 2013); H2 occurs in symbiosis with *Breviolum minutum* (referred to as SSB01, Xiang et al., 2013), whereas CC7 associates with *Symbiodinium linucheae* (referred to as SSA01, Sunagawa et al., 2009b; Bieri et al., 2016) and RS with *Symbiodinium microadriaticum* (Cziesielski et al., 2018). Additionally, a new host-symbiont combination was introduced by re-infecting aposymbiotic CC7 individuals with SSB01 Symbiodiniaceae (Baumgarten et al., 2015), herein referred to as CC7-SSB01. Animals were reared in the laboratory as previously described (Röthig et al., 2016a; Herrera et al., 2017); in clear polycarbonate tanks (2 L capacity; Cambro Camwear, Huntington Beach, CA, United States) filled with autoclaved natural seawater collected from the RS (∼39 PSU salinity and pH ∼8) under white-light (∼80 μmol photons m^–2^ s^–1^ of photosynthetically active radiation) on a 12 h light: 12 h dark cycle, and fed with freshly hatched *Artemia* brine shrimp twice per week. Further, anemones were cultured at 25°C (control culture conditions) and 32°C (heat stress treatment). All tanks were maintained under the same conditions, except for temperature, for at least 2 years before performing experiments.

### 16S rRNA Gene Sequencing

Briefly, a total of 48 samples (3 anemones × 2 replicate tanks × 4 host-symbiont combinations × 2 temperatures, Supplementary Figure S1) were processed according to Röthig et al. (2016a) and Herrera et al. (2017). DNA extractions were performed following the DNeasy Plant Mini Kit (Qiagen, Hilden, Germany) manufacturer’s instructions. Regions V5 and V6 of the 16S rRNA gene were amplified using the primers 784F and 1061R (Andersson et al., 2008) with Illumina (San Diego, CA, United States) adapter overhangs. We sequenced the V5-V6 hypervariable regions because these have been shown to have superior phylogenetic resolution for bacterial phyla (Yang et al., 2016) and are more closely aligned with the SILVA database classifier (Zhang et al., 2018). Indeed, many studies target these regions in particular to examine bacterial diversity in corals (Röthig et al., 2016b; Ziegler et al., 2016; Hadaidi et al., 2017) and Aiptasia (Röthig et al., 2016a; Herrera et al., 2017). For each sample, PCRs were done in triplicate using a Qiagen Multiplex PCR kit (Qiagen, Hilden, Germany) with a final primer concentration of 0.6 μM in a 15 μL final reaction volume. Thermocycling conditions were as follows: an initial activation step of 15 min at 95°C, 27 cycles each of 30 s at 95°C, 90 s at 55°C, and 30 s at 72°C, followed by a final extension step of 10 min at 72°C. Triplicate PCRs were then pooled for each sample, cleaned using Agencourt AMPure XP magnetic beads (Beckman Coulter, Indianapolis, IN, United States) and subsequently indexed with Nextera XT barcoded sequencing adapters (Illumina, San Diego, CA, United States). Libraries were quantified using a QuBit dsDNA BR Kit (Thermo Fisher Scientific, Waltham, MA, United States), pooled in equimolar ratios and run on a 2% agarose gel to isolate and purify the final library (MinElute Gel Extraction Kit; Qiagen, Hilden, Germany). Finally, amplicon length and quality were checked on a BioAnalyzer (Agilent Technologies, Santa Clara, CA, United States) before sequencing on the Illumina MiSeq platform (2 × 300 bp, paired-end v3 chemistry) according to the manufacturer’s instructions.

### Microbial Data Processing

A total of 6,765,347 reads were demultiplexed and adapter sequences were removed with MiSeq Reporter (v.2.4.60.8; Illumina, San Diego, CA, United States). Data was analyzed using the software MOTHUR (Schloss et al., 2009) following the same procedures described in Röthig et al. (2016a) and Herrera et al. (2017). Reads were assembled into 6,765,347 contigs, trimmed and quality-filtered [i.e., sequences with ambiguous bases, long homopolymers (>5) or insufficient length were removed]. Singletons were removed (1,931,982) and the remaining sequences were aligned against the SILVA database (release 119, Pruesse et al., 2007) and pre-clustered based on 2 bp differences (Huse et al., 2010). Chimeras were removed (113,695) using VSEARCH (Rognes et al., 2016), as were other unwanted sequences (i.e., sequences assigned to chloroplasts, mitochondria, archaea, and eukaryotes). Only sequences that were phylogenetically classified as bacteria (Greengenes release gg_13_8_99; bootstrap = 60, McDonald et al., 2012) were used for further analyses. From the resulting 3,090,432 sequences (average length of 293 bp) further subsampling of 15,028 sequences per sample was done. As shown by a rarefaction analysis (Supplementary Figure S2), this was sufficient to capture the majority of the bacterial diversity. Finally, sequences were clustered into Operational Taxonomic Units (OTUs) using a 97% similarity cutoff. Noteworthy, taxonomic annotation errors have been reported when using the Greengenes database, especially for the orders Vibrionales and Alteromonadales (Edgar, 2018; Lydon and Lipp, 2018). Thus, for those OTUs of interest, representative sequences were BLASTed against NCBI’s GenBank nr to identify previous occurrences of identical or highly similar bacterial sequences (i.e., first best match based on sequence identity and query cover criteria). For the purpose of comparing our results to other studies (Röthig et al., 2016a; Brown et al., 2017; Herrera et al., 2017; Ziegler et al., 2017) both taxonomic annotations were reported, but if different from each other, only the latter was considered in further analyses.

### Data Analysis

Methods for analyzing microbiome diversity have been extensively reviewed (Knights et al., 2011; Goodrich et al., 2014); thus standardizing diversity measurements in terms of within (i.e., alpha) and between samples (i.e., beta). First, we examined differences in the bacterial communities within each Aiptasia group across treatments by calculating α-diversity indices (as implemented in MOTHUR). Data was tested for normal distribution using the Shapiro–Wilk test and analyzed via two-way analysis of variance (ANOVA) or two-factorial generalized linear models (GLMs) fitted with gamma distribution and “log” link function (if non-parametric), using host-symbiont combination and temperature as explanatory variables. An overview of the models is provided in the Supplementary Table S2. Significant differences were then identified via *post hoc* comparisons. Further, permutational multivariant analysis of variance (PERMANOVA, Anderson, 2017) tests (as implemented in the R package *vegan*, Oksanen et al., 2019) were performed for all Aiptasia groups and temperatures. Differentially abundant OTUs were then determined using the package DESeq2 (Love et al., 2014), which uses a negative binomial distribution model that takes into account sample library size and dispersion for each taxon. Wald test *p*-values were adjusted with the Benjamini–Hochberg correction method.

β-diversity was also analyzed to better understand the effect of temperature, host-symbiont combination and batch (i.e., technical variation) in shaping the microbiome of Aiptasia. Generally speaking, β-diversity metrics can be quantitative (i.e., using sequence abundance) or qualitative (i.e., considering only presence-absence of sequences), and they can be based on phylogeny or not. While phylogeny-based metrics might outperform other measures in community-level comparisons, the latter can still be useful in clustering analyses. Specific methods used to cluster samples (both distance metrics and clustering algorithms) can affect the outcome and its interpretation. Thus, it is of extreme importance to perform clustering using several different approaches to ensure that sample classification (clustering) is not dependent on a particular parameter (Kuczynski et al., 2010; Goodrich et al., 2014). β-diversity was first analyzed by computing UniFrac dissimilarity matrices (Lozupone et al., 2011) based on the OTU table and phylogenetic tree produced by MOTHUR. Principal coordinates analysis (PCoA) plots were used to visualize distances between samples and thus evaluate the effect of temperature on the bacterial communities of Aiptasia. Changes in the composition and structure of the community were studied using unweighted (i.e., purely based on sequence distances) and weighted UniFrac (i.e., includes both sequence and abundance information) methods, respectively. This was performed first for all the dataset together so that overall patterns could be inspected, and then for each group separately to avoid any confounding effects resulting from the biological variation between different host-symbiont combinations. Dispersion effects (i.e., based on sample distance to the centroid of each group) of the different groups and treatments were quantified by conducting permutation tests of multivariate dispersion (PERMDISP, Anderson, 2008) as implemented in R using the package *vegan* (Oksanen et al., 2019). Additional clustering analyses were performed using non-phylogenetic metrics such as Bray-Curtis, Pearson and Kendall Tau methods, also widely used in community ecology, to calculate and plot pairwise β-diversity distances (custom Python script available in https://github.com/lyijin/anna_karenina). Ward’s clustering criteria was applied. Different approaches have different merits (Parks and Beiko, 2013; Weiss et al., 2016). Whilst Bray-Curtis measures abundance similarity, the Pearson method evaluates the degree of linear dependence between two variables and Kendall measures how well this relationship can be described using a monotonic function, for example. Therefore, in order to catch all possible correlations, all three methods were simultaneously used.

## Results

Here, we investigated the effect of long-term heat stress (>2 years) on the bacterial communities of four different Aiptasia host-symbiont combinations; H2 (associated with its native symbiont *B. minutum*), CC7 (naturally occurring with *S. linucheae*), RS (symbiotic with *S. microadriaticum*) and CC7-SSB01, an experimental combination resulting from re-infecting aposymbiotic CC7 with *B. minutum* originally from H2. We assessed changes in the microbiota by analyzing within- and between- (Aiptasia group and temperature) diversity, and explored β-diversity patterns using different clustering methods. Moreover, our study offers another level of reproducibility by considering batch effects.

### Dysbiotic Microbiota Are More Diverse

**FIGURE 1:**
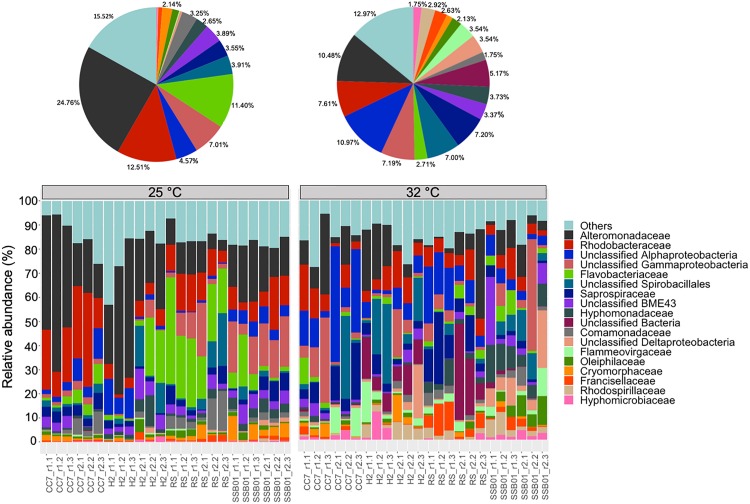
Bacterial community composition of different Aiptasia host-symbiont combinations on the family level (Greengenes database, bootstrap ≥ 60). Each color represents one taxon across all samples. Less abundant taxa <1%), comprising 199 distinct families are grouped under “others.” Pie charts display the average composition of anemones at 25°C (left) and 32°C (right). Sample labels indicate Aiptasia group, batch (i.e., tank) and replicate, respectively (e.g., CC7_r1.1).

**FIGURE 2:**
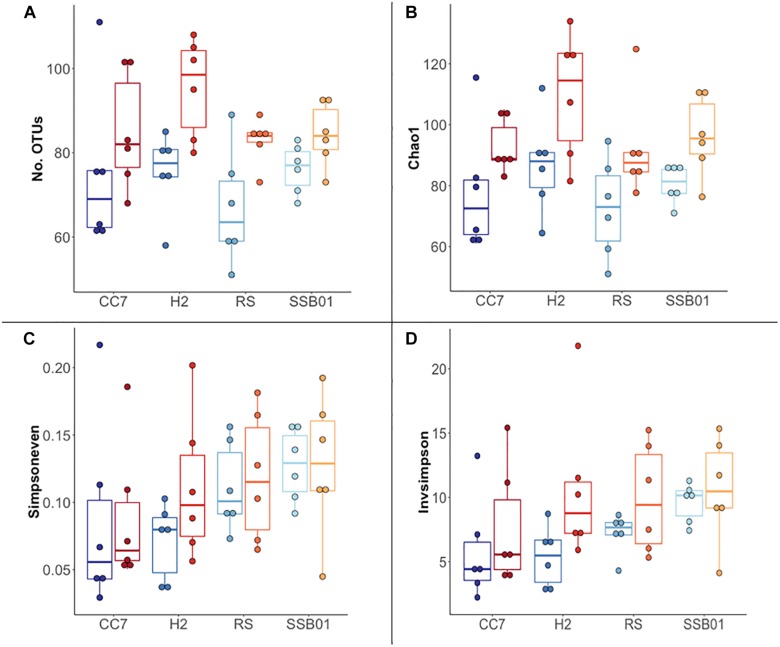
Overview of α-diversity measurements for different Aiptasia host-symbiont combinations. Number of observed OTUs **(A)**, Chao1 richness estimator **(B)**, Inverse Simpson **(C)**, and Simpson’s evenness **(D)** indexes. Each boxplot shows six data points corresponding to the replicate samples of each host-symbiont combination. Different shades of blue and red represent 25°C and 32°C temperatures, respectively.

**FIGURE 3:**
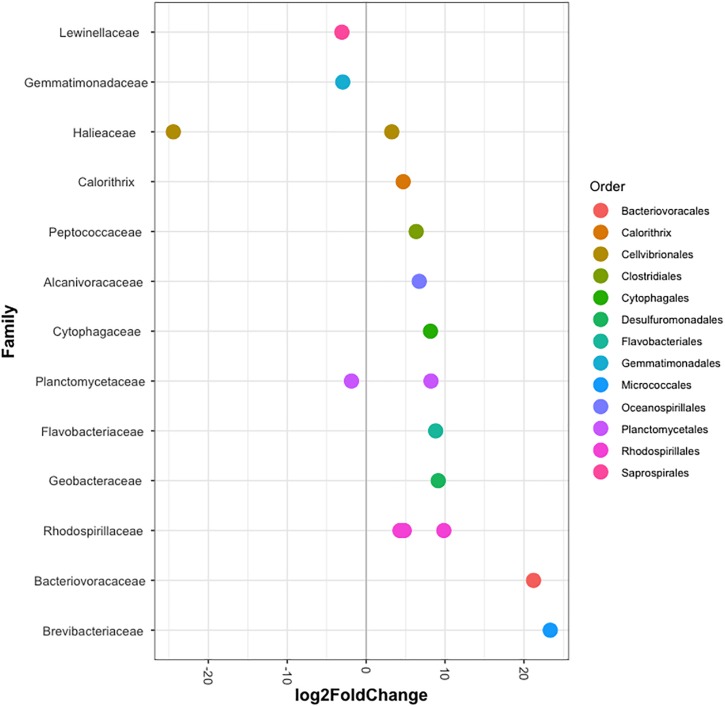
Differentially abundant OTUs (agglomerated by family) in Aiptasia between temperatures. Log2 fold change values indicate OTUs that show significant differential abundance in anemones reared at 32°C compared to those from 25°C (*p* < 0.05). Taxonomic annotation shown is based on BLASTn closest hit.

The bacterial communities of all host-symbiont combinations and temperatures were examined at the family level (Figure 1). Variations within each Aiptasia group (i.e., batch effects corresponding to the two replicate tanks from which anemones were taken) were detected but not significant (*p*_PERMANOVA_ > 0.05 for all comparisons). Overall, Aiptasia from 32°C had a higher number of taxa (1088) than those reared at 25°C (1037). Members of the families Alteromonadaceae (10.48%) and Rhodobacteraceae (7.61%) were found in all the Aiptasia examined here, albeit in lower abundances compared to 25°C (24.76 and 12.51%, respectively). Notably, taxa belonging to Rhodospirillaceae and Flammeovirgaceae were also more abundant (100% presence) in anemones cultured at 32°C. Accordingly, OTU richness and diversity were also higher in this treatment (Figure 2 and Supplementary Table S1). All α-diversity estimators were significantly different between temperatures except for the number of observed OTUs and the Inverse Simpson index (Supplementary Table S2). Moreover, from a total of 724 OTUs, the genus *Glaciecola* sp. (family Alteromonadaceae) was the most dominant at both temperatures. Further analysis of raw abundances showed 17 differentially abundant OTUs, from which 13 were over and 4 under represented at 32°C compared to 25°C, respectively (Figure 3 and Supplementary Table S3). Notably, OTUs belonging to the family Rhodospirillaceae, Planctomycetaceae, and Cytophagaceae were significantly more abundant in heat-stressed anemones compared to Aiptasia reared at 25°C. Whilst, at the same time, OTUs annotated to the orders Actinomycetales and Oceanospirillales were significantly decreased in the bacterial communities of 32°C (Figure 3).

### Temperature Effects on β-Dispersion

**FIGURE 4:**
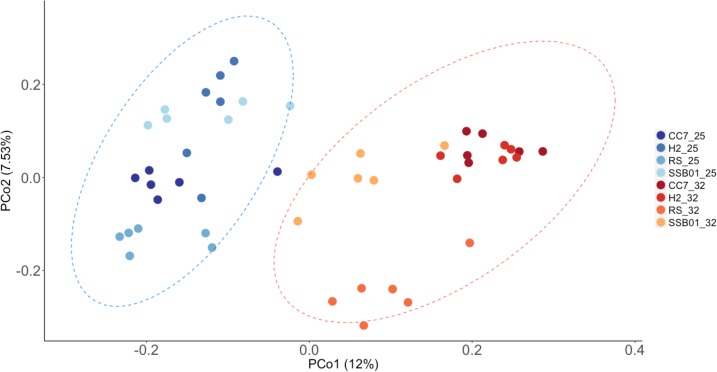
Principal coordinate analysis based on unweigthed Unifrac distances showing changes in the bacterial community composition of Aiptasia across temperatures. Different shades of blue and red show 25°C and 32°C, respectively. Ellipses represent 95% confidence intervals. Percentages on axes indicate the variation explained by the two coordinates.

Significant differences in the overall composition (unweighted UniFrac, Figure 4) and structure (weighted UniFrac, Supplementary Figure S3) of the microbiota were observed. This was further substantiated by PERMANOVA analyses, which showed a clear separation between temperature (*p* = 0.001) and Aiptasia groups (CC7 *p* = 0.006, H2 *p* = 0.004, RS *p* = 0.002, and SSB01 *p* = 0.004). Moreover, anemones reared at 32°C also exhibited increased β-diversity dispersion in their bacterial consortia (*p* = 0.003, PERMDISP based on Bray-Curtis distances). Overall, bacterial communities were more scattered (i.e., higher dispersion, Supplementary Figure S4) in heat-stressed Aiptasia. However, when analyzed separately for each group, only CC7 was significant (*p* = 0.033).

### Temperature Effect on Shaping the Bacterial Composition of Aiptasia

Overall, cluster analyses revealed temperature, and not host-symbiont combination or batch effect, as the main factor driving differences in the bacterial composition of Aiptasia (Supplementary Figure S5). Although there were no clear patterns, differences between treatments were still discernible. Both Bray-Curtis (Supplementary Figure S5A) and Pearson (Supplementary Figure S5B) methods separated the data in two main clusters corresponding to samples from 32°C and 25°C, respectively. Interestingly, no clear distinction between temperature treatments was observed with Kendall’s rank-order correlations (Supplementary Figure S5C). Instead, all RS Aiptasia were clustered together, suggesting this particular host-symbiont combination might have a distinct microbiome composition compared to the other Aiptasia groups. Nonetheless, different methods are based on different assumptions (and constrains), and so data interpretation can vary. Finally, analyses also showed in all cases that individuals first clustered according to the tank they were sampled from, and then by host-symbiont combination and treatment. Thus, highlighting the presence of batch effects (but as mentioned before, not significant).

## Discussion

The structure and function of animals’ microbial communities are strongly affected by the environment (Allison and Martiny, 2008; Shade et al., 2012), therefore, having a better understanding of the effects of environment on the associated microbial communities is a key goal in microbial ecology (Green et al., 2008). Many studies have shown that variations in environmental parameters such as temperature (Sunagawa et al., 2015), salinity (Székely et al., 2013), pH (Liu et al., 2015), and organic compounds (Dang and Lovell, 2016) shape the microbial composition and structure of different ecosystems. Stressors in general can change a healthy stable microbiome to a dysbiotic unstable state (Teplitski et al., 2016; McDevitt-Irwin et al., 2017). The mechanisms by which this transition occurs are, however, still poorly understood. Regardless, research has shown that changes in the coral-associated microbial communities can ultimately disrupt holobiont functioning (Thompson et al., 2015; Bourne et al., 2016; Peixoto et al., 2017), thus serving as a diagnostic tool for assessing the health of corals (Glasl et al., 2017).

Questions such as whether the type and magnitude of microbial taxa changes over space and time (Glasl et al., 2018), or how it differs between healthy and diseased ecosystems (Hoisington et al., 2015; Zaneveld et al., 2016; Wang et al., 2017) can be answered by quantifying bacterial community diversity. Richness (i.e., total number of species) and evenness (i.e., proportional abundances of species) are some of the taxon-based measures commonly applied to describe the diversity within a given community (α-diversity). Likewise, the turnover of species between groups (β-diversity) is used to characterize the biological diversity among communities and along environmental gradients (Lozupone and Knight, 2008). One or the other alone cannot describe all aspects of a community’s diversity, especially since taxon-based methods ignore the fact that species are not equally related, thus masking important patterns in the data (Lozupone and Knight, 2008). Instead, divergence-based methods that take into account phylogenetic distance (e.g., Unifrac measures, Lozupone et al., 2011) can provide a better insight into the community structure. These metrics, in conjunction, can then be used to examine variability and stability of the microbiome (Zaneveld et al., 2017).

### Distinct Bacterial Microbiomes of Aiptasia Reared at Different Temperatures

Overall, the bacterial composition of all Aiptasia host-symbiont combinations examined here was more diverse in the high temperature (32°C) treatment. Our results showed increased richness (i.e., the total number of observed OTUs) and α-diversity (i.e., the total number of species and their relative abundances) in response to long-term temperature stress. This is not surprising as it has already been shown that corals’ microbial diversity is higher not only in response to increasing seawater temperatures (Tout et al., 2015; Welsh et al., 2015; Lee et al., 2016) but also to reduced pH (Meron et al., 2011), water pollution (Ziegler et al., 2016), and disease (Kimes et al., 2010).

In context, the ability of disturbed corals to regulate or reject incoming bacteria from the environment may be reduced, thus resulting in a higher number of taxa (McDevitt-Irwin et al., 2017). Yet, the opposite can also occur (Meron et al., 2012; Tracy et al., 2015; Morrow et al., 2017). Stress in humans’ gut microbiome, for example, allows for opportunistic bacteria to dominate the microbial community, which in turn leads to a lower α-diversity (Lozupone et al., 2012). Although it is not clear to which degree single strains of bacteria play a role in the tolerance or susceptibility of corals to environmental stressors, meta-analyses of 16S data have suggested that certain taxa in particular are opportunistic and potentially pathogenic thus being highly present in diseased individuals (Teplitski et al., 2016; McDevitt-Irwin et al., 2017). Accordingly, in this study we found that taxa belonging to the families Alteromonadaceae, Flammeovirgaceae, Rhodobacteraceae and Rhodospirillaceae were highly abundant at 32°C compared to 25°C (Figure 1). Three OTUs annotated to Rhodospirillaceae were significantly more abundant in heat-stressed anemones (Figure 4). Similarly, OTUs from the classes Cytophagia and Planctomycetia were also increased at 32°C compared to 25°C. Previous studies in corals have shown that these particular taxonomic groups increase at higher temperatures (McDevitt-Irwin et al., 2017; Ziegler et al., 2017). The family Alteromonadaceae has also been shown to be associated with stressed or diseased corals and has been identified as an age indicator, even though members of this family are also known to reside in healthy corals (Sunagawa et al., 2009a; Glasl et al., 2016). Moreover, Flammeovirgacea has been shown to significantly increase in abundance throughout the coral mucus aging cycle (Sweet and Bulling, 2017). The most dominant taxa in both treatments, although mostly increased at 25°C, was *Glaciecola* sp. (OTU001), a genus that has already been reported in corals from hot environments (temperatures up to 33°C, Ziegler et al., 2017). This genus is particularly known for having genomic features related to cold adaptation (Qin et al., 2013), and has therefore been linked to thermal tolerance (Ziegler et al., 2017).

### Stressed Microbiomes Are More Variable

Only recently, β-diversity dispersion measures have been applied in microbiome studies to show destabilization of the microbiome and bacterial opportunism in stressed and diseased corals (Zaneveld et al., 2016). Here, we observed increased β-diversity and dispersion of Aiptasia bacterial communities in response to temperature stress. Noteworthy, salinity of the seawater used to rear Aiptasia in this study (∼39 PSU from the central Red Sea) is significantly higher than from other geographic regions. High salinity levels have been shown to have a great impact on corals’ microbiome stability (Röthig et al., 2016b). Thus, it is important to take this into account, as salinity is an additional abiotic factor that might confound the effect of temperature we observed in this study. Regardless of not having clear patterns (i.e., cluster analysis) an overall separation between treatments was still observed, supporting the notion that temperature was the main driver of bacterial community differences.

The changes in the bacterial composition observed in this study are in line with the recently described “Anna Karenina principle” for animal microbiomes coined by Zaneveld et al. (2017), which postulates that external stressors often cause a more stochastic (i.e., randomly distributed) community structure due to the host being unable to regulate its microbiome when disturbed. Patterns consistent with this principle have been observed in corals but also in higher organisms like humans (David et al., 2014; Wang et al., 2017). It is interesting, however, that this concept does not hold for sponge microbiomes (Glasl et al., 2018). Moreover, even if this principle has been confirmed in some cases, it still has not been fully established if the observed increase in diversity in response to stress is a common biological phenomenon or rather a result of bias introduced by sampling (i.e., samples taken at different time points might reflect different states of a dynamic microbiome restructuring process). Consequently, comparison of microbiomes sampled at different time points during a dynamic restructuring process would suggest an increase of diversity. This study, however, compared microbiomes of hosts subjected to 2 years of constant heat stress. Hence, it can be assumed that the microbiomes have reached a final stable state and that the observed increase in β-diversity is not due to differences in sampling time. It is then fair to say that Aiptasia falls within this pattern, as increased microbiome β-diversity and dispersion was observed (i.e., greater distances between data points and centroid, Supplementary Figure S4) for individuals reared at higher temperature.

### Temperature Shapes the Bacterial Community of Aiptasia

The microbiota of animals is complex, it is shaped through host–specific interactions (e.g., microbial recognition mechanisms and host immune responses, Ding et al., 2016; Neave et al., 2016b), interactions between members of the community (e.g., viruses and fungi, and Symbiodiniaceae in the case of corals (Deines and Bosch, 2016; Duerkop, 2018), geographical location and environmental conditions (Hong et al., 2009; Ceh et al., 2010). Several studies in different coral species have shown how the environment can have a greater impact than the host genetics in driving and shaping the microbiome (Littman et al., 2009; Pantos et al., 2015; Roder et al., 2015; Rothschild et al., 2018). Consistent species-specific associations still occur as it has been suggested for the widespread and highly abundant *Endozoicomonas* symbionts, found in many corals with contrasting life-history traits and across global scales, for example (Neave et al., 2016a). In Aiptasia, studies showed that the bacterial associations of H2 and CC7 Aiptasia lines cultured under identical laboratory conditions are significantly different, suggesting a species-specific microbiota (Röthig et al., 2016a; Herrera et al., 2017). Although the microbiome of Aiptasia from the Red Sea has still not been described, it is not surprising that these individuals clustered in a separate group (Supplementary Figure S5C). As the Red Sea displays unique physicochemical conditions, so are the microbial communities of corals and epilithic biofilms that thrive in these environments (Roik et al., 2016, 2019; Röthig et al., 2016b; Hadaidi et al., 2017). Thus, studying the bacterial communities from this region may provide a model for understanding the dynamics and functioning of coral reefs under predicted “future ocean” scenarios.

Finally, our study shows that even if host-symbiont combination has a strong effect on the microbiome composition (as shown in the PCoAs and cluster analyses), temperature is the dominating factor structuring the bacterial communities of Aiptasia. Furthermore, we also observed variations within each Aiptasia group, even if all individuals examined here were reared and sampled under the same conditions. This variation most probably stems from batch effects (i.e., replicate tanks), which calls for attention when looking at the reproducibility of microbiome studies. Technical sources of variation are often overlooked even though they are very important to control for statistically, otherwise biased data can lead to potentially erroneous conclusions.

## Conclusion

Host-associated microbial partnerships in coral reef ecosystems is a growing field of study, and for this purpose the sea anemone Aiptasia has served as a cnidarian-Symbiodiniaceae model system. We believe that studying the effect of heat stress on the bacterial communities of Aiptasia is a start to better comprehend the microbial dynamics and resilience of this and other holobionts like stony corals. Our data showed that all Aiptasia host-symbiont combinations reared at different temperatures harbored distinct bacterial compositions. Moreover, we found that the bacterial consortia associated with heat-stressed individuals exhibited increased β-diversity and dispersion. The latter led us to explore the recently proposed AKP for disturbed microbiomes, which aims toward a better understanding of host resilience, as the microbiome status can greatly influence the animal fitness and health.

## Ethics Statement

This study was exempt due to the use of invertebrate animals.

## Author Contributions

MH and MA designed and conceived the experiments. MA contributed with reagents, materials, and analysis tools. HIA and MH generated the data. HIA, MH, YJL, and MA analyzed and interpreted the data. HIA and MH wrote the manuscript with input from all authors. All authors read and approved the final manuscript.

## Conflict of Interest Statement

The authors declare that the research was conducted in the absence of any commercial or financial relationships that could be construed as a potential conflict of interest.
