# Erratum: Long-Term Temperature Stress in the Coral Model Aiptasia Supports the “Anna Karenina Principle” for Bacterial Microbiomes

**DOI:** 10.3389/fmicb.2019.01709

**Published:** 2019-07-18

**Authors:** Frontiers Production Office

**Affiliations:** Frontiers Media SA, Lausanne, Switzerland

**Keywords:** β-diversity, dispersion, microbiome, 16S rRNA gene, temperature

Due to an editorial error, the Data Availability Statement containing the raw sequences was erroneously omitted from the article. The publisher apologizes for the mistake.

## Data Availability

All sequence data from this study has been deposited on NCBI under BioProject accession no. PRJNA497709 (https://www.ncbi.nlm.nih.gov/sra/?term=PRJNA497709).

