# Supplementary material for: Long-Term Temperature Stress in the Coral Model Aiptasia Supports the “Anna Karenina Principle” for Bacterial Microbiomes
Source: Front Microbiol. 2019 May 8;10:975. doi: 10.3389/fmicb.2019.00975 (PMC6517863; doi:10.3389/fmicb.2019.00975)
Supplement: Supplementary file 1 [file Image_1.pdf]

**Supplementary Materials**

**Supplementary figures**

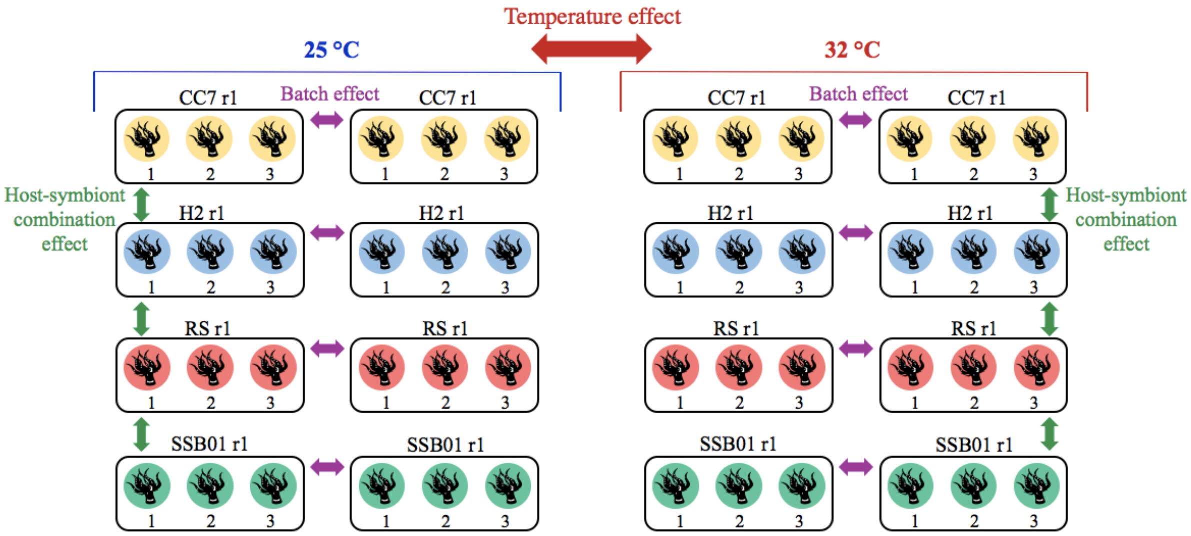


**Figure S1.** Illustration of experimental schematic shows 2 temperature groups (25 °C and 32 °C) to assess the temperature effect on the bacterial compositions. Each temperature group has 4 Aiptasia host-symbiont combination which allow to reveal the strain effect, within each strain there are 2 tanks (r1 and r2) were anemones sampled from (i.e., batch effect).

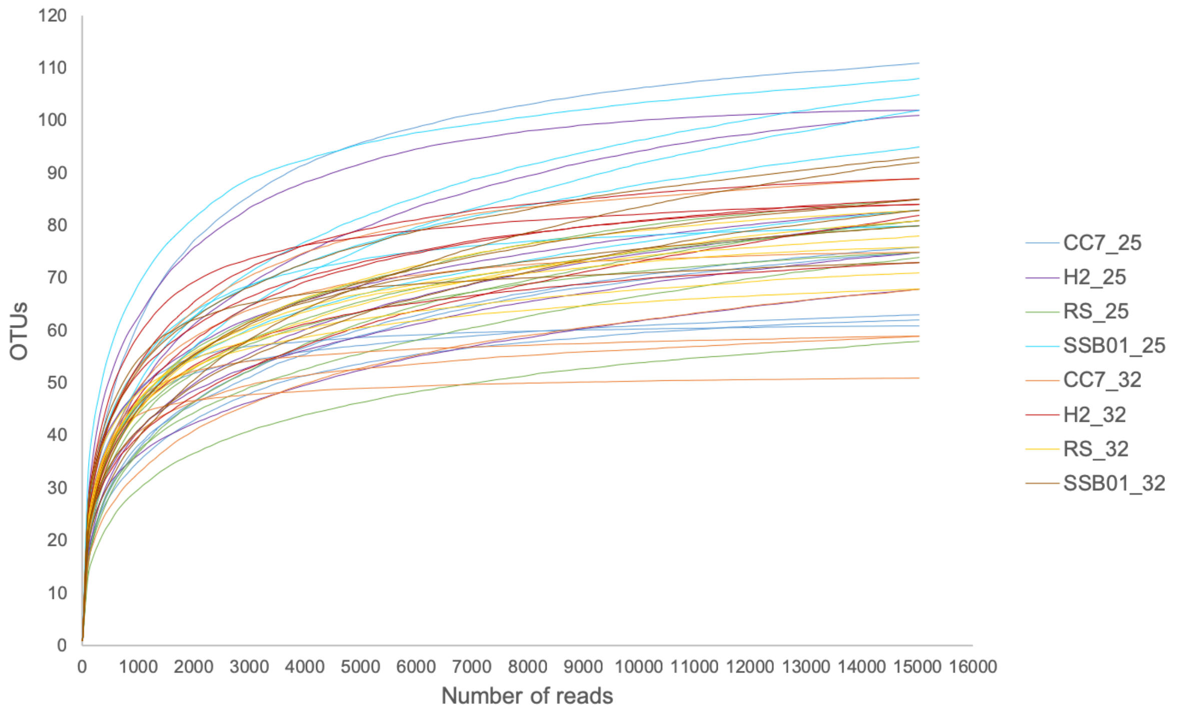


**Figure S2**. Individual rarefaction curves showing OTU richness as a function of sequencing depth for the subsampled dataset (n = 15,028 sequences per sample). The curve becomes asymptotic as the OTU number saturates, indicating a sufficient coverage for each sample analyzed. OTUs were defined at 97% similarity level.

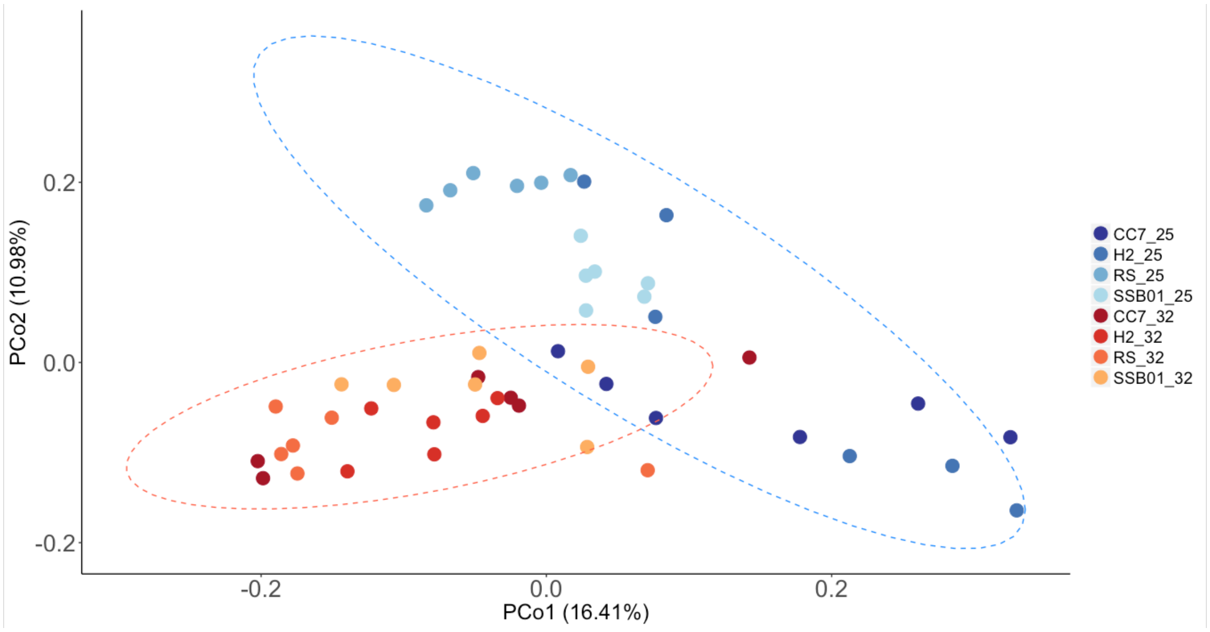


**Figure S3.** Principal coordinate analysis based on weighted Unifrac distances showing Aiptasia bacterial communities are different between temperatures. Different shades of blue and red show 25 °C and 32 °C respectively. Ellipses represent 95% confidence intervals. Percentages on axes indicate the variation explained by the two coordinates.

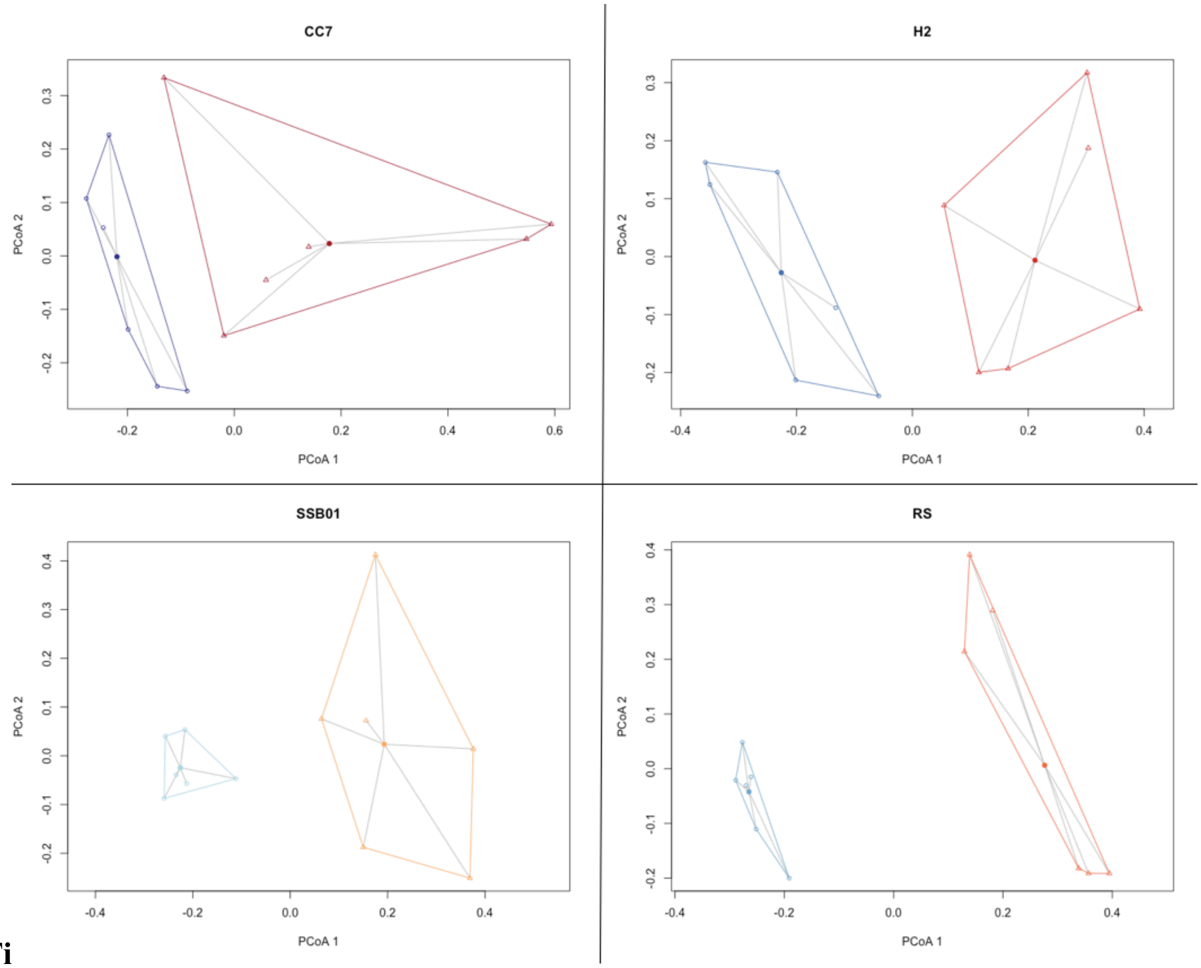


**Fi**

**Figure S4.** β–diversity dispersion of the bacterial communities of different Aiptasia host-symbiont combination (permutation test for homogeneity of multivariate dispersions based on Bray-Curtis distances): CC7 **(A)**, H2 **(B)**, RS **(C)** and SSB01 **(D)**. Different shades of blue and red data points correspond to individuals from 25 °C and 32 °C respectively.

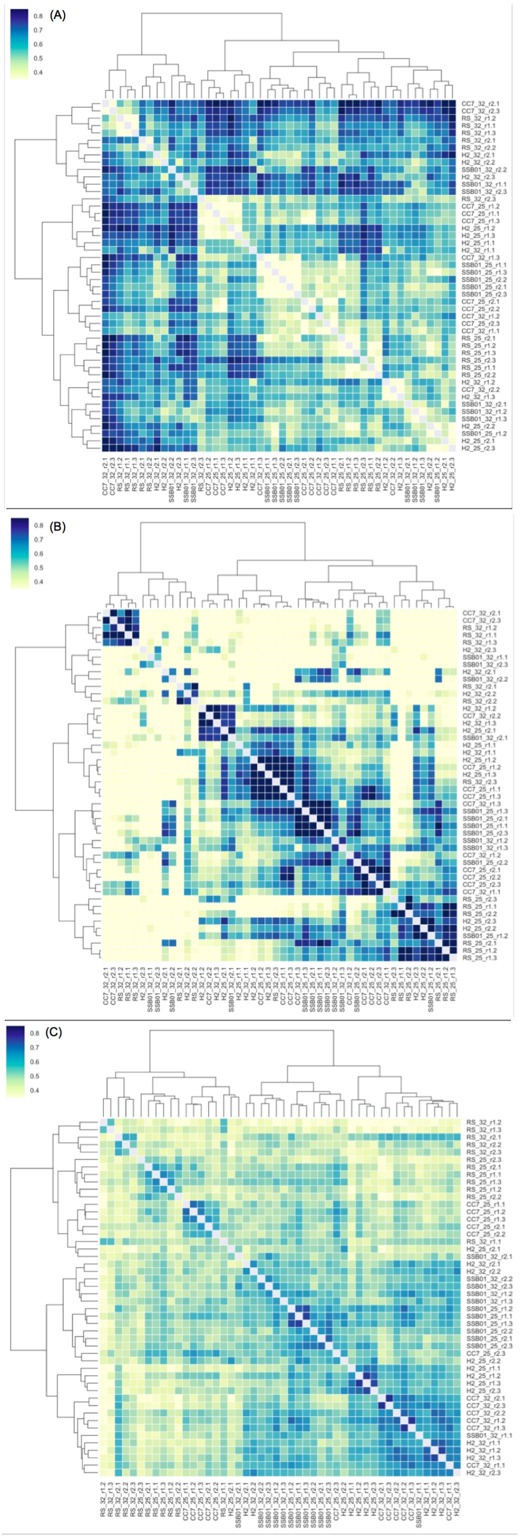


**Figure S5.** Cluster analyses based on pairwise beta-diversity values using: Bray-Curtis dissimilarity matrix (A), and Pearson (B) and Kendall-Tau (C) correlation coefficients. Ward's method was used for the clustering. Sample labels indicate Aiptasia group, batch (i.e., tank) and replicate, respectively (e.g., CC7_r1.1)
