# Supplementary material for: Long-Term Temperature Stress in the Coral Model Aiptasia Supports the “Anna Karenina Principle” for Bacterial Microbiomes
Source: Front Microbiol. 2019 May 8;10:975. doi: 10.3389/fmicb.2019.00975 (PMC6517863; doi:10.3389/fmicb.2019.00975)
Supplement: Supplementary file 2 [file Table_1.doc]

**Supplementary Materials**

**Supplementary tables**

**Table S1.** Summary of alpha diversity measures (mean ± SD) of 16S rRNA gene sequencing

of the bacterial communities associated with different laboratory-cultured Aiptasia host-symbiont combinations at 25 °C and 32 °C.

|  | **Groups** | **No. OTUs** | **Chao1** | **Invsimpson** | **Simpsoneven** |
| --- | --- | --- | --- | --- | --- |
| 25 °C | CC7 | 74.67 (±19) | 77.94 (±20.43) | 5.8 (±3.99) | 0.09 (±0.07) |
|  | H2 | 75.5 (±9.48) | 86.79 (±15.86) | 5.38 (±2.33) | 0.07 (±0.03) |
|  | RS | 66.83 (±13.66) | 72.73 (±16.25) | 7.22 (±1.54) | 0.11 (±0.03) |
|  | SSB01 | 76.17 (±5.78) | 80.58 (±6.2) | 9.62 (±1.51) | 0.13 (±0.03) |
| 32 °C | CC7 | 85 (±13.81) | 92.74 (±8.86) | 7.6 (±4.66) | 0.09 (±0.05) |
|  | H2 | 95.5 (±11.71) | 109.88 (±20.53) | 10.66 (±5.84) | 0.11 (±0.05) |
|  | RS | 82.83 (±5.34) | 92.18 (±16.72) | 9.91 (±4.22) | 0.12 (±0.05) |
|  | SSB01 | 84.33 (±7.53) | 96.28 (±13.13) | 10.6 (±4.04) | 0.13 (±0.05) |

**Table S2**. Overview of model results for statistical analysis of individual alpha diversity

indices. Number of observed OTUs (A) and Chao1 (B) were analyzed in two-way analysis of variance (ANOVA) using host-symbiont combination and temperature as dependent variables. InvSimpson (C) and Simpsoneven (D) were analyzed in a generalized linear model (GLM) with γ-distribution, also using host-symbiont combination and temperature as the dependent variables, since data did not adhere to a normal distribution.

| **A** – **No. observed OTUs** (ANOVA) | | *Df* | *F* | *p* | |
| --- | --- | --- | --- | --- | --- |
| Host-symbiont combination | | 3 | 6.559 | **<0.001** | |
| Temperature | | 1 | 2.387 | **0.129** | |
| **B** – **Chao1** (ANOVA) | | *Df* | *F* | *p* | |
| Host-symbiont combination | | 3 | 2.527 | **0.070** | |
| Temperature | | 1 | 17.648 | **<0.001** | |
| **C** – **InvSimpson** (GLM, γ-distr.) | | *n* | *𝜒^2^* | *p* | |
| Host-symbiont combination | | 47 |  |  | |
| Temperature | | 47 | 1.974 | **0.055** | |
| **D** – **Simpsoneven** (GLM, γ-distr.) | | *n* | *𝜒^2^* | *p* | |
| Host-symbiont combination | | 47 |  |  | |
| Temperature | | 47 | 2.316 | **<0.05** | |
|  | | | |  |  |
